# Visualizing the valence states of europium ions in Eu-doped BaAl_2_O_4_ using X-ray nanoprobe mapping

**DOI:** 10.1107/S1600577521012947

**Published:** 2022-01-18

**Authors:** Yu-Hao Wu, Yung-Yang Lin, Jeng-Lung Chen, Shih-Yu Fu, Shu-Chi Huang, Chien-yu Lee, Bo-Yi Chen, Gung-Chian Yin, E-Wen Huang, Mau-Tsu Tang, Bi-Hsuan Lin

**Affiliations:** a Department of Materials Science and Engineering, National Yang Ming Chiao Tung University, Hsinchu 30010, Taiwan; b National Synchrotron Radiation Research Center, Hsinchu 30076, Taiwan

**Keywords:** X-ray nanoprobes, XEOL, XRF, XAS, phosphor materials

## Abstract

This study develops and successfully demonstrates visualization methods for the characterization of europium (Eu)-doped BaAl_2_O_4_ phosphors using X-ray nanoprobe techniques. X-ray fluorescence (XRF) mapping not only gives information on the elemental distributions but also clearly reveals the valence state distributions of the Eu^2+^ and Eu^3+^ ions. The accuracy of the estimated valence state distributions was examined by performing X-ray absorption spectroscopy (XAS) across the Eu *L*_3_-edge (6.977 keV). The X-ray excited optical luminescence (XEOL) spectra exhibit different emission lines in the selected local areas. Their corresponding emission distributions can be obtained via XEOL mapping. The emission properties can be understood through correlation analysis. The results demonstrate that the main contribution to the luminescence intensity of the Eu-doped BaAl_2_O_4_ comes from the Eu^2+^ activator and the emission intensity will not be influenced by the concentration of Eu^2+^ or Eu^3+^ ions. It is anticipated that X-ray nanoprobes will open new avenues with significant characterization ability for unravelling the emission mechanisms of phosphor materials.

## Abstract 2

It is anticipated that X-ray nanoprobes will open new avenues with significant characterization ability for unravelling the emission mechanisms of phosphor materials.

## 1. Introduction

Phosphor-converted white-light-emitting diodes (wLEDs) have been studied widely to develop more stable and efficient wLEDs (Xia *et al.*, 2019 ▸). Investigating the emission mechanism of long-afterglow phosphors will help realize various applications in lighting. Alkaline earth aluminates are good candidates for host matrices for luminescent materials, and are widely used as hosts for rare earths. Persistently luminescent phosphors composed of alkaline earth aluminates can be represented by the general formula *X*Al_2_O_4_ (*X* = Mg^2+^, Ca^2+^, Sr^2+^ or Ba^2+^) (Lephoto *et al.*, 2012 ▸). Recently, the widely used traditional host matrix BaAl_2_O_4_ has received extensive attention because of its unique properties, such as low cost, easy synthesis, high physical stability and better luminescence efficiency (Yin *et al.*, 2020 ▸; Lephoto *et al.*, 2012 ▸; Tian *et al.*, 2021 ▸; Rezende *et al.*, 2012 ▸, 2016 ▸, 2015 ▸).

Many researchers have extensively investigated methods to obtain perfect luminescent phosphors. For example, Tian *et al.* (2021 ▸) reported a new BaAl_2_O_4_–YAG:Ce composite ceramic phosphor for high-efficiency wLEDs. Yin *et al.* (2020 ▸) reported a BaAl_2_O_4_:Eu^2+^–Al_2_O_3_ composite ceramic to enhance the luminescence output. Rafiaei *et al.* (2020 ▸) reported the synthesis, crystal structure and optical and adsorption properties of BaAl_2_O_4_:Eu^2+^, Eu^2+^/*L* ^3+^ (*L* = Dy, Er, Sm, Gd, Nd, and Pr) phosphors. As BaAl_2_O_4_ doped with rare earths has significant application in luminescent phosphors, in this report we propose a powerful analysis method to help researchers improve their manufacturing methods.

Rare earth ions play the most important roles as activators in these phosphors. In particular, the valence states of the rare earth ions significantly affect the emission properties of the phosphors. In the case of a europium (Eu)-doped BaAl_2_O_4_ phosphor, a broad-band emission at ∼500 nm can be attributed to the Eu^2+^ emissions (4*f*5*d* → 4*f* transitions), and the narrow emission peaks at around 560–750 nm are associated with the Eu^3+^ emissions (^5^ *D*_0_ → ^7^ *F*_*i*_, *i* = 0 to 4). X-ray nanoprobe (Sham, 2014 ▸; Martínez-Criado *et al.*, 2014 ▸; Lin *et al.*, 2020 ▸) techniques using a synchrotron source can be applied to characterize these phosphors (Huang *et al.*, 2021 ▸). By exploiting the advantages of X-ray nanoprobes, including the continuously tunable X-ray energy (4–15 keV) and excellent spatial resolution of the nano-focused X-ray beam (<100 nm), we can easily and quickly investigate the valence states of the rare earth ions in the selected local area to unravel the emission mechanisms of phosphor materials. An X-ray nanoprobe can be used to perform X-ray absorption spectroscopy (XAS) across the *L*-edges of Eu (6.977 keV), Dy (7.790 keV), Er (8.358 keV), Sm (6.716 keV), Gd (7.243 keV), Nd (6.208 keV) and Pr (5.964 keV) to obtain information on the valence states.

In this study, we developed visualization methods for the characterization of the Eu-doped BaAl_2_O_4_ phosphor. Using an X-ray nanoprobe, X-ray fluorescence (XRF) and X-ray excited optical luminescence (XEOL) mapping can clearly reveal the distributions of the constituent elements, the valence states of the Eu^2+^ and Eu^3+^ ions, and the different emission wavelengths (λ_em_). The accuracy of the estimated valence state distributions was examined using XAS spectra. As the X-ray nanoprobe can provide excellent spatial resolution, we selected different local areas with different valence states of the Eu^2+^ and Eu^3+^ ions to study their emission properties. The XEOL spectra consist of one broad intense peak at ∼500 nm and narrow emission peaks at around 560–750 nm in the local areas richer in Eu^2+^ and Eu^3+^, respectively. In addition, a weaker emission at ∼390 nm, which is related to the F colour centre of α-Al_2_O_3_, is observed.

## 2. Experiment

The XAS, XRF and XEOL experiments were conducted on the Taiwan Photon Source (TPS) 23A X-ray nanoprobe beamline located at the National Synchrotron Radiation Research Center (NSRRC) in Taiwan. The capabilities of the TPS 23A X-ray nanoprobe beamline have previously been described in detail by Lin *et al.* (2019 ▸, 2020 ▸). This beamline can deliver an X-ray beam spot size of less than 60 nm. The test powder sample of Eu-doped BaAl_2_O_4_ (Ba_0.97_Eu_0.03_Al_2_O_4_) was doped with Eu^2+^ and Eu^3+^ ions and purchased from Dott Technology. The XAS and XRF spectra were measured using a silicon drift detector (SDD; Vortex-ME4, Hitachi). The XEOL spectra were collected using a multimode optical fibre (with a core diameter of 400 µm) attached to a spectrometer (iHR320, Horiba) with a deep thermoelectric cooling charge-coupled device (Syncerity BI UV-Vis) and a resolution of 2048 × 70 pixels. The XEOL mapping images were acquired through a photomultiplier tube, which is installed in another spectrometer (iHR550, Horiba).

## 3. Results

**Figure 1:**
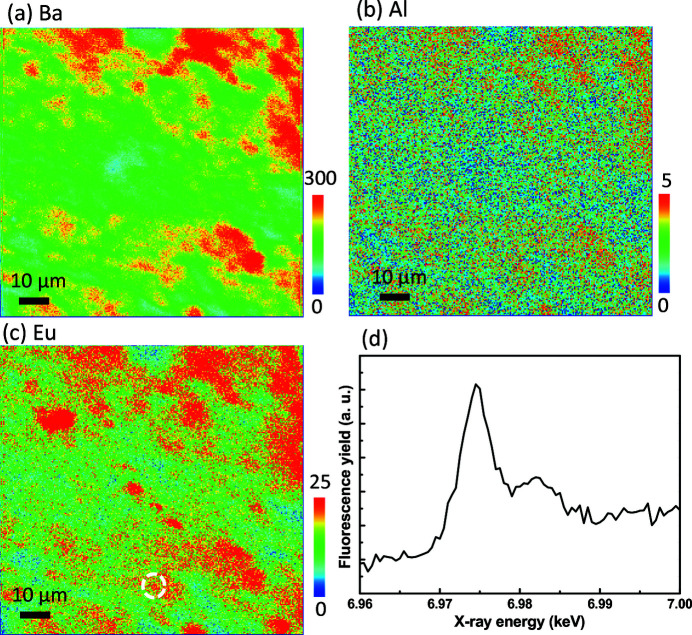
XRF maps of Eu-doped BaAl_2_O_4_, showing the elemental distributions of (*a*) Ba, (*b*) Al and (*c*) Eu. (*d*) X-ray absorption spectrum of the location marked with the white dashed circle in panel (*c*). On the basis of features of the Eu^2+^ and Eu^3+^ ions, XAS can reveal the corresponding amounts of the Eu^2+^ and Eu^3+^ ions.

The mapping images used to visualize the Eu-doped BaAl_2_O_4_ phosphor, shown in Figs. 1 ▸–3 ▸ ▸, have the same measured areas. Through XRF and XEOL mapping, detailed information about the measured area can be obtained. Fig. 1 ▸ shows the XRF and XAS analyses of the Eu-doped BaAl_2_O_4_ phosphor. As the X-ray energy was tuned at 6.985 keV, which is above the Ba *L*_3_-edge (5.247 keV), Al *K*-edge (1.560 keV) and Eu *L*_3_-edge (6.977 keV), the elemental distributions of Ba, Al and Eu were observed directly, as shown in Figs. 1 ▸(*a*), 1 ▸(*b*) and 1 ▸(*c*), respectively. The Eu *L*_3_-edge XAS spectra exhibit a strong white line caused by the electronic transitions from 2*p*_3/2_ to 5*d*. In particular, the resonances of the Eu^2+^ and Eu^3+^ ions are located at 6.975 and 6.983 keV, respectively.

The core electrons in the Eu^3+^ ions have a larger binding energy than those in the Eu^2+^ ions, with the difference between the resonance energies equal to ∼8 eV (Korthout *et al.*, 2013 ▸). On the basis of features of the Eu^2+^ and Eu^3+^ ions, XAS can reveal the corresponding amounts of the Eu^2+^ and Eu^3+^ ions. Fig. 1 ▸(*d*) shows the XAS spectrum of the area marked with the white dashed circle in Fig. 1 ▸(*c*). The XAS spectrum of this local area shows that the fluorescence yield of the Eu^2+^ resonance (6.975 keV) is higher than that of the Eu^3+^ resonance (6.983 keV), which indicates that the highlighted local area is richer in Eu^2+^.

Although the elemental distribution of Eu can be obtained from Fig. 1 ▸(*c*), it cannot provide information on the positions of the Eu^2+^ and Eu^3+^ ions. Therefore, we developed a visualization method that directly images the valence states of the Eu ions. As the X-ray energy of the synchrotron source is continuously tunable, we selected three X-ray energies at 6.970, 6.975 and 6.983 keV for XRF mapping; these correspond to the background, Eu^2+^ resonance and Eu^3+^ resonance, respectively. Using the following equations, we subsequently obtained the distributions of the valence states of the Eu ions,

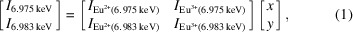




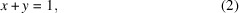




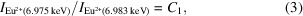




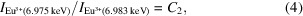




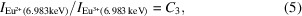




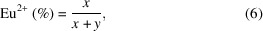




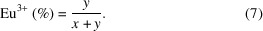




Assuming that the concentrations of the Eu^2+^ and Eu^3+^ ions are *x*% and *y*%, respectively, the fluorescence yields of *I*_6.975 keV_ and *I*_6.983 keV_ can be estimated using equation (1). The corresponding ratio parameters *C*_1_, *C*_2_ and *C*_3_ were determined from the XAS spectra of the reference materials for Eu^2+^ (EuS) and Eu^3+^ (Eu_2_O_3_) (Korthout *et al.*, 2013 ▸). In the report by Korthout *et al.*, the Eu *L*_3_-edge XAS spectra of the EuS and Eu_2_O_3_ reference materials show that the values of 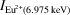 , 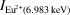 , 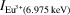 and 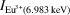 were 1.9, 1, 0.36 and 2.1, respectively. So, the ratio parameters *C*_1_, *C*_2_ and *C*_3_ can be calculated to be equal to 1.9, 0.17 and 0.476, respectively.

**Figure 2:**
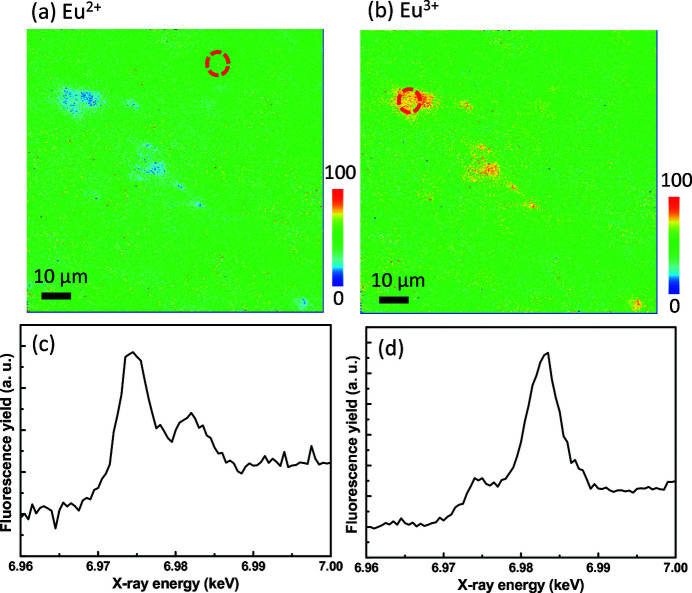
Estimation results for the distributions of (*a*) Eu^2+^ and (*b*) Eu^3+^ ions. Panels (*c*) and (*d*) show XAS spectra of the locations/areas marked with the red dashed circles in panels (*a*) and (*b*), respectively. The XAS spectra demonstrate the accuracy of the estimated results, implying that the valence states of the Eu ions can be easily and quickly visualized using XRF mapping.

After estimating the above parameters, we obtained the distributions of the Eu^2+^ and Eu^3+^ ions using equations (6) and (7), as shown in Figs. 2 ▸(*a*) and 2 ▸(*b*), respectively. To verify the accuracy of the estimated results, we obtained the XAS spectra of the selected local areas to measure the valence states of the Eu ions. Fig. 2 ▸(*c*) shows the XAS spectrum of the area marked with the red dashed circle in Fig. 2 ▸(*a*). The XAS spectrum in Fig. 2 ▸(*c*) shows that the fluorescence yield of the Eu^2+^ resonance (6.975 keV) is higher than that of the Eu^3+^ resonance (6.983 keV), indicating that this local area is richer in Eu^2+^. According to our estimation, another local area marked with the red dashed circle in Fig. 2 ▸(*b*) is clearly richer in Eu^3+^. Fig. 2 ▸(*d*) illustrates the XAS spectrum of the local area marked in Fig. 2 ▸(*b*), which is consistent with the expected results. In Fig. 2 ▸(*d*), it can be observed that the fluorescence yield of the Eu^2+^ resonance (6.975 keV) is lower than that of the Eu^3+^ resonance (6.983 keV), indicating that this local area is richer in Eu^3+^.

The XAS spectra demonstrate the accuracy of the estimated results, implying that the valence states of the Eu ions can be easily and quickly visualized using XRF mapping. This model relies on the approximation that EuS and Eu_2_O_3_ are used as the reference for Eu^2+^ and Eu^3+^, respectively, in the composite. Since the difference between the resonance energies of Eu^2+^ and Eu^3+^ is ∼8 eV, the Eu *L*_3_-edge XAS can easily reveal the corresponding amounts of Eu^2+^ and Eu^3+^ ions. It may increase the discrepancy while the difference between the resonance energies is smaller.

Although this model can provide the relative quantitative values of Eu^2+^ and Eu^3+^, the validity of the quantitative values of the valence states can be measured by XAS spectra. In the quantitative analysis reported by Yamamoto & Yukumoto (2018 ▸), the oxidation state of Eu in phosphor samples was established using XAS to investigate the influence of the constituent elements, absorption edge and measurement mode on the evaluated oxidation states.

**Figure 3:**
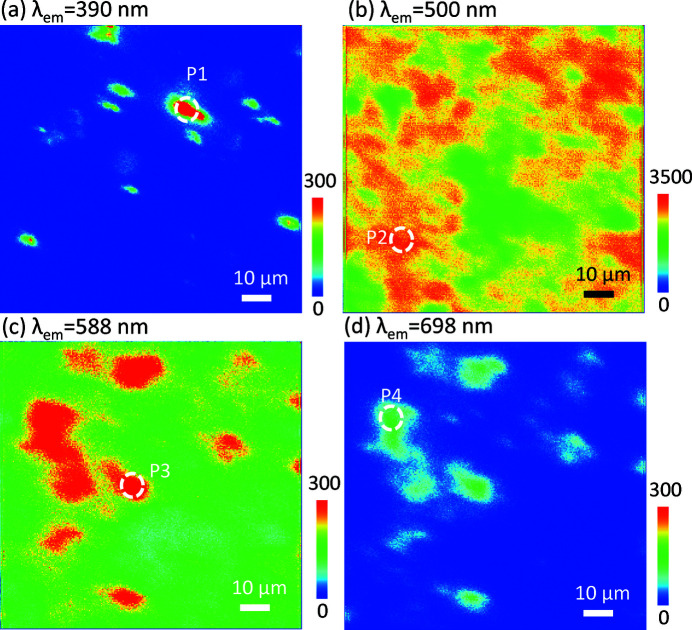
XEOL mapping performed at emission wavelengths (λ_em_) of approximately (*a*) 390, (*b*) 500, (*c*) 588 and (*d*) 698 nm. The emission distributions of the Eu-doped BaAl_2_O_4_ phosphor at different emission wavelengths (λ_em_) are clearly visualized.

The variation in the valence states of the Eu ions tends to influence the emission properties. Because the Eu^2+^ and Eu^3+^ ions have different distributions, as shown in Fig. 2 ▸, we used XEOL mapping to study further the emission distribution of the main emission wavelength of the Eu-doped BaAl_2_O_4_ phosphor. Figs. 3 ▸(*a*), 3 ▸(*b*), 3 ▸(*c*) and 3 ▸(*d*) show the XEOL mapping at emission wavelengths (λ_em_) of approximately 390, 500, 588 and 698 nm, respectively. The emission distributions of the Eu-doped BaAl_2_O_4_ phosphor at different emission wavelengths (λ_em_) were clearly visualized. At λ_em_ = 500 nm, the well documented emission of the Eu^2+^ ions due to the 4*f*5*d* → 4*f* transitions was observed, along with emissions of the Eu^3+^ ions at λ_em_ = 588 and 698 nm due to transitions of the ^5^ *D*_0_ → ^7^ *F*_*i*_ (*i* = 0 to 4) states (Rezende *et al.*, 2016 ▸). However, we suggest that λ_em_ = 390 nm is attributed to the F centre of α-Al_2_O_3_, which will be discussed later. Thus, XEOL mapping can be used to visualize clearly the emission behaviour of the Eu-doped BaAl_2_O_4_ phosphor. Compared with Figs. 3 ▸(*a*), 3 ▸(*c*) and 3 ▸(*d*), Fig. 3 ▸(*b*) shows a larger emission intensity and better emission uniformity. This result demonstrates that the main contribution to the luminescence intensity of Eu-doped BaAl_2_O_4_ comes from the Eu^2+^ activator.

**Figure 4:**
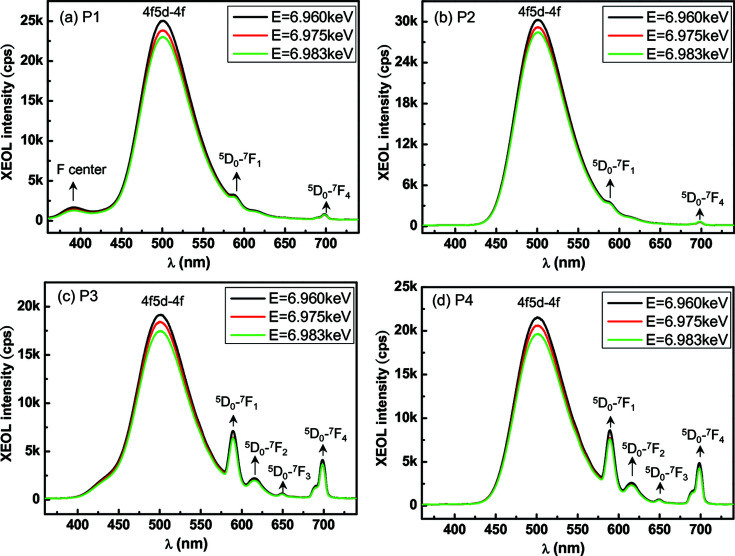
(*a*)–(*d*) Room-temperature XEOL spectra of the four local areas marked with the white dashed circles in Figs. 3 ▸(*a*)–3 ▸(*d*). The XEOL spectra corresponding to each of the four local areas were acquired at three X-ray energies: below the Eu *L*_3_-edge (6.960 keV), at the Eu^2+^ resonance (6.975 keV) and at the Eu^3+^ resonance (6.983 keV).

To study further the emission properties of the Eu-doped BaAl_2_O_4_ phosphor in different local areas, we selected four such local areas, indicated by the white dashed circles in Figs. 3 ▸(*a*)–3 ▸(*d*), and plotted the corresponding XEOL spectra. The room-temperature XEOL spectra of the four local areas (P1–P4) for excitation across the Eu *L*_3_-edge (6.977 keV) are shown in Figs. 4 ▸(*a*)–4 ▸(*d*).

The XEOL spectra corresponding to each of the four local areas were acquired at three X-ray energies: below the Eu *L*_3_-edge (6.960 keV), at the Eu^2+^ resonance (6.975 keV) and at the Eu^3+^ resonance (6.983 keV). The XEOL spectra of P1–P4 exhibit one common broad intense peak at 500 nm, which can be attributed to the Eu^2+^ 4*f*5*d* → 4*f* transitions.

The above results not only reinforce the conclusion that the Eu^2+^ activator dominates the luminescence intensity in the Eu-doped BaAl_2_O_4_ phosphor, but also illustrate that the Eu^2+^ ions have unique local symmetry in the matrix. According to Rezende *et al.* (2011 ▸, 2016 ▸), Eu^2+^ ions may be incorporated on more than one non-symmetric site, as substitution might occur at either the Al^3+^ or Ba^2+^ site, which results in more than one band in the emission spectra associated with the 4*f*5*d* → 4*f* transition. Another shoulder emission due to the doping of Eu^2+^ ions on the non-equivalent sites can be observed in the intense peak. In addition, the emission intensity of the single intense peak in P1–P4 decreased slightly as the X-ray energy was tuned across the Eu *L*_3_-edge. This behaviour suggests that the energy transfer to the Eu^2+^ 4*f*5*d* → 4*f* transitions is less efficient above than below the Eu *L*_3_-edge (Huang *et al.*, 2021 ▸).

In addition to the single intense peak at ∼500 nm produced by the Eu^2+^ 4*f*5*d* → 4*f* transitions, the XEOL spectra of P1–P4 exhibit other weaker peaks. The XEOL spectra of P1 shown in Fig. 4 ▸(*a*) consist of a special weaker emission line at ∼390 nm. This emission may be attributed to the F-centre emission in α-Al_2_O_3_ (den Engelsen, Fern, Ireland & Silver, 2020 ▸). The F-centre is a type of colour centre that is associated with an oxygen vacancy with two electrons (Wang *et al.*, 2013 ▸; Ghamnia *et al.*, 2003 ▸; Itou *et al.*, 2009 ▸). den Engelsen, Fern, Ireland, Yang & Silver (2020 ▸) also observed an F-centre emission in the Eu-doped BaAl_2_O_4_ phosphor via photoluminescence and cathodoluminescence.

The XEOL spectra of P2 shown in Fig. 4 ▸(*b*) consist of only one intense peak produced by the 4*f*5*d* → 4*f* transitions of Eu^2+^ and very weak peaks associated with the ^5^ *D*_0_ → ^7^ *F*_*i*_ (*i* = 0 to 4) transitions of the Eu^3+^ ions, showcasing the perfect luminescence intensity of the Eu-doped BaAl_2_O_4_ phosphor. Compared with P1 and P2, the XEOL spectra of local areas P3 and P4 clearly consist of emission peaks associated with the ^5^ *D*_0_ → ^7^ *F*_*i*_ (*i* = 0 to 4) transitions of the Eu^3+^ ions, as shown in Figs. 4 ▸(*c*) and 4 ▸(*d*), respectively. Clearly, the emission lines caused by the Eu^3+^ transitions in P3 and P4 are narrower than those in P1 and P2, indicating more crystal homogeneity in local areas P3 and P4 than in P1 and P2. The broad Eu^3+^ emission is a result of crystal inhomogeneity causing small distortions around the Eu ions (Rezende *et al.*, 2016 ▸; Gasparotto *et al.*, 2008 ▸).

**Figure 5:**
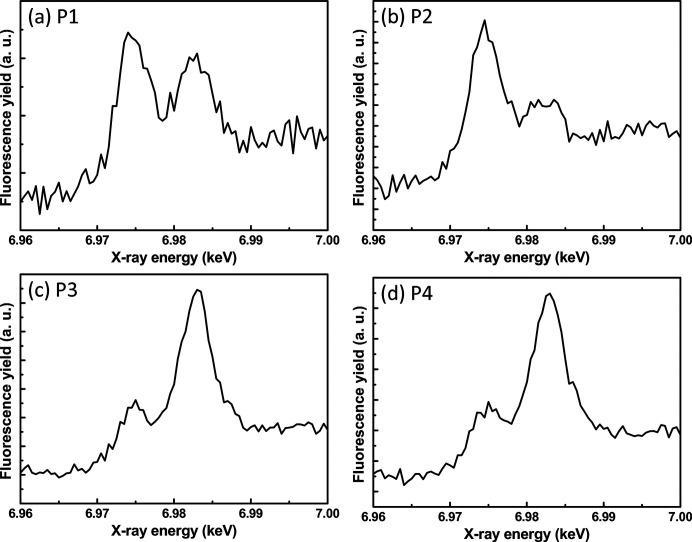
(*a*)–(*d*) XAS spectra of local areas P1–P4 marked with the white dashed circles in Figs. 3 ▸(*a*)–3 ▸(*d*). The coexistence of the two common valence states of Eu^2+^ and Eu^3+^ can be seen in the XAS spectra of P1–P4, represented by the two well resolved edge resonances.

To study the valence states of the Eu ions, the same four local areas P1–P4 were used to measure the Eu *L*_3_-edge XAS spectra, as shown in Figs. 5 ▸(*a*)–5 ▸(*d*). The coexistence of the two common valence states of Eu^2+^ and Eu^3+^ can be seen in the XAS spectra of P1–P4, represented by the two well resolved edge resonances. Although the Eu^2+^ and Eu^3+^ ions coexist, the corresponding concentrations of the Eu^2+^ and Eu^3+^ ions in these local areas can be determined from the fluorescence yield of the XAS spectra. The XAS spectrum of P1 shows that the fluorescence yield of the Eu^2+^ resonance is similar to that of the Eu^3+^ resonance, indicating similar concentrations of the Eu^2+^ and Eu^3+^ ions in local area P1. However, the XAS spectrum of P2 shows that the fluorescence yield of the Eu^2+^ resonance is higher than that of the Eu^3+^ resonance, indicating that local area P2 is richer in Eu^2+^. Compared with P1 and P2, the XAS spectra of P3 and P4 show the opposite behaviour, that is, the Eu^3+^ resonances have a higher fluorescence yield and therefore local areas P3 and P4 are richer in Eu^3+^.

The results of the XAS spectra are consistent with those of the XEOL spectra, as shown in Fig. 5 ▸. Because local area P2 is richer in Eu^2+^, the XEOL spectra of P2 consist of only one intense peak at ∼500 nm produced by the Eu^2+^ 4*f*5*d* → 4*f* transitions. As P3 and P4 are richer in Eu^3+^, their XEOL spectra consist of narrow emission lines around 580–700 nm that are produced by the ^5^ *D*_0_ → ^7^ *F*_*i*_ (*i* = 0 to 4) transitions of the Eu^3+^ ions.

**Figure 6:**
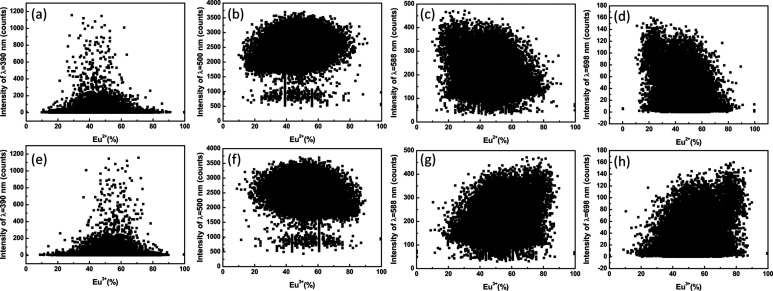
Rows (*a*)–(*d*) and (*e*)–(*h*) show the emission intensities of λ_em_ = 390, 500, 588 and 698 nm as a function of Eu^2+^ and Eu^3+^ ions, respectively. A pixel-by-pixel analysis can be conducted to determine the overall correlation between the λ_em_ = 390, 500, 588 and 698 nm and Eu^2+^ and Eu^3+^ ions.

Since we can obtain the distributions of the Eu^2+^ and Eu^3+^ ions from the results of Figs. 2 ▸(*a*) and 2 ▸(*b*), a pixel-by-pixel analysis can be conducted to determine the overall correlation between λ_em_ = 390, 500, 588 and 698 nm and Eu^2+^ and Eu^3+^ ions. For a given map, λ_em_ emission intensity and Eu ions were plotted against each other for each pixel in the map. Figs. 6 ▸(*a*)–6 ▸(*d*) and 6 ▸(*e*)–6 ▸(*h*) show the emission intensity of λ_em_ = 390, 500, 588 and 698 nm as a function of Eu^2+^ and Eu^3+^ ions, respectively. The correlations are consistent with the measured results of the XEOL and XAS spectra. The emission intensity of λ_em_ = 390 nm shown in Figs. 6 ▸(*a*) and 6 ▸(*e*) has largest emission intensity at around 50% Eu^2+^ or Eu^3+^ ions, suggesting that P1 has similar concentrations of Eu^2+^ and Eu^3+^ ions. Figs. 6 ▸(*b*) and 6 ▸(*f*) show that the emission intensity of λ_em_ = 500 nm is a zero correlation with Eu^2+^ or Eu^3+^ ions. This suggests that, regardless of whether local areas P1–P4 are richer or poorer in Eu^2+^ or Eu^3+^ ions, the single intense peak at ∼500 nm is still the main contribution to the luminescence intensity. The emission intensities of λ_em_ = 588 and 698 nm show negative correlation with the Eu^2+^ ions shown in Figs. 6 ▸(*c*) and 6 ▸(*d*), and positive correlation with the Eu^3+^ ions shown in Figs. 6 ▸(*g*) and 6 ▸(*h*). This result is also corroborated by the fact that the local areas P3 and P4 are richer in Eu^3+^. Thus, the emission mechanisms of Eu-doped BaAl_2_O_4_ phosphors can be further understood through such a correlation analysis.

## 4. Conclusions

In this paper, we report powerful characterization capabilities for investigating the features of Eu-doped BaAl_2_O_4_ phosphor materials using an X-ray nanoprobe. XRF and XEOL mapping can provide clear visualization images containing detailed distribution information on Eu-doped BaAl_2_O_4_ phosphors, including the elements, the valence states of the Eu ions and the different emission wavelengths (λ_em_). The accuracy of the estimated valence state distributions was examined by performing XAS across the Eu *L*_3_-edge (6.977 keV), and the corresponding concentrations of the Eu^2+^ and Eu^3+^ ions were obtained from the XAS spectra.

Exploiting the excellent spatial resolution of the X-ray nanoprobe, we selected four local areas with different valence states of the Eu^2+^ and Eu^3+^ ions to study their emission properties. The XEOL spectra consisted of one broad intense peak at ∼500 nm and narrow weaker emission peaks at around 560–750 nm in the local areas richer in Eu^2+^ and Eu^3+^, respectively. In addition, a weaker emission at ∼390 nm relating to the F colour centre of α-Al_2_O_3_ was also observed.

The XEOL spectra demonstrated that the main contribution to the luminescence intensity of Eu-doped BaAl_2_O_4_ comes from the Eu^2+^ activator and the emission intensity will not be influenced by the concentration of Eu^2+^ or Eu^3+^ ions.

We believe that X-ray nanoprobes will open new avenues with significant characterization ability for unravelling the emission mechanisms of phosphor materials.
